# No evidence for morphometric associations of the amygdala and hippocampus with the five-factor model personality traits in relatively healthy young adults

**DOI:** 10.1371/journal.pone.0204011

**Published:** 2018-09-20

**Authors:** Joshua C. Gray, Max M. Owens, Courtland S. Hyatt, Joshua D. Miller

**Affiliations:** 1 Department of Medical and Clinical Psychology, Uniformed Services University, Bethesda, MD, United States of America; 2 Department of Psychology, University of Georgia, Athens, Georgia, United States of America; University of Pennsylvania, UNITED STATES

## Abstract

Despite the important functional role of the amygdala and hippocampus in socioemotional functioning, there have been limited adequately powered studies testing how the structure of these regions relates to putatively relevant personality traits such as neuroticism. Additionally, recent advances in MRI analysis methods provide unprecedented accuracy in measuring these structures and enable segmentation into their substructures. Using the new FreeSurfer amygdala and hippocampus segmentation pipelines with the full Human Connectome Project sample (N = 1105), the current study investigated whether the morphometry of these structures is associated with the five-factor model (FFM) personality traits in a sample of relatively healthy young adults. Drawing from prior findings, the following hypotheses were tested: 1) amygdala and hippocampus gray matter volume would be associated with neuroticism, 2) CA2/3 and dentate gyrus would account for the relationship of the hippocampus with neuroticism, and 3) amygdala gray matter volume would be inversely associated with extraversion. Exploratory analyses were conducted investigating potential associations between all of the FFM traits and the structure of the hippocampus and amygdala and their subregions. Despite some previous positive findings of whole amygdala and hippocampus with personality traits and related psychopathology (e.g., depression), the current results indicated no relationships between the any of the brain regions and the FFM personality traits. Given the large sample and utilization of sophisticated analytic methodology, the current study suggests no association of amygdala and hippocampus morphometry with major domains of personality.

## Introduction

Personality–“relatively enduring styles of thinking, feeling, and acting”[1]–is consistently associated with morbidity and mortality [2], well-being [3], occupational functioning [4], relational functioning [5], antisocial behavior and aggression [6], and forms of psychopathology (e.g., [7,8]) including anxiety depression, addiction, and personality disorders. Personality is shaped by both genetic and environmental factors [9] and exhibits a relatively similar structure across cultures [1,10]. While several overlapping taxonomies exist that posit slightly fewer (e.g., [11,12]) or more dimensions (e.g., [13]), the big five/five-factor model (FFM) of personality [14] is arguably the most widely used taxonomy. This model posits five primary domains of personality–neuroticism, extraversion, openness, agreeableness, and conscientiousness–that are related meaningfully to a host of important outcomes [15].

Due to the broad public health relevance of the FFM (e.g., [16]), researchers have increasingly sought to understand the biological basis of these traits. For example, genetics research has yielded multiple genome-wide significant variants of FFM traits (for a review see [17]). Likewise, numerous functional magnetic resonance imaging (fMRI) studies have linked FFM traits to patterns of brain activity during relevant tasks and during rest (e.g., [18,19]). Finally, a number of studies have explored the structural neuroanatomy underlying the FFM.

Unfortunately, many of the neuroanatomical results have been inconsistent or conflicting, due to variation in methods (e.g., only focusing on certain FFM traits, type of MRI analyses used) and limited sample sizes. In an effort to resolve many of these discrepancies, Riccelli et al. (2017) recently used data from the Human Connectome Project (N = 507) to conduct the largest and most comprehensive study of the cortical correlates of the FFM to date [20]. Among the findings most convergent with the second largest study by Bjørnebekk et al. (2013) (N = 265) [21] was the association of neuroticism with smaller area in prefrontal-temporal regions. However, key differences have been found as well; for instance, the former study found extraversion was associated with posterior regions like the precuneus, superior temporal gyrus, entorhinal cortex, and fusiform gyrus [20], whereas the latter found only an association with thinner inferior frontal gyrus [21]. In general, Riccelli et al. (2017) found numerous associations across all FFM traits that had not been previously reported. Indeed, just as in genetics research [17], increasing sample sizes enables the accurate assessment of smaller effect sizes in morphometric research.

In addition to cortical correlates of the FFM, the amygdala and hippocampus have been studied in relation to extraversion, neuroticism, and conscientiousness, due to their role in emotional and social processing [22–27]. Despite theoretical justification and fMRI studies suggesting important relationships between FFM traits and these regions (e.g., [18,19]), the relationships in morphometric studies are inconsistent to date (Table 1). With regard to extraversion, one early study found a positive association between extraversion and left amygdala gray matter density (but not volume) [28], another found a positive association with right amygdala volume [29], but a third study found a negative association with bilateral amygdala volume [30], and four others found no relationship [31–34]. With regard to neuroticism, a meta-analysis found negative emotionality (including studies of neuroticism) was associated with a larger left amygdala and a qualitative review found no clear pattern of association with the hippocampus [35]. Additionally, the largest single study of neuroticism and morphometry of the amygdala (N = 1050) found that neuroticism was associated with increased bilateral amygdala volume [36]. Only one study has explored the association of the hippocampus with extraversion and conscientiousness, and no direct associations were identified [34]. In sum, the research indicates the amygdala has no clear relationship with extraversion and appears to be positively associated with neuroticism. The limited number of studies of the hippocampus in relation to the FFM traits, neuroticism and extraversion in particular, is surprising considering its role in emotional responses, connectivity with the amygdala [37], and association with multiple of psychiatric disorders [38–40].

**Table 1:** Previous studies that assessed amygdalar and hippocampal volume associations with FFM.

Study	Software	Region	Participants^1^	Age	Extraversion	Neuroticism	Conscientiousness
Omura et al., 2005	SPM	Amygdala	41	23.8	Null	Null	—
Wright et al., 2006	FreeSurfer	Amygdala	28	24.0	Null	Null	—
Wright et al., 2007	FreeSurfer	Amygdala	29	70.3	Null	Null	—
Cremers et al., 2011	SPM	Amygdala	65	40.5	Increased R amygdala volume	Null	—
Jackson et al., 2011	FreeSurfer	Amygdala and hippocampus	79	66.0	Null	Null	Null
Holmes et al., 2012	FreeSurfer	Amygdala	1050	21.4	—	Increased L and R amygdala volume	—
Koelsch et al., 2013	SPM	Amygdala	59	24.2	Null	Increased L amygdala volume	—
Lu et al., 2014	SPM	Amygdala	71	22.4	Reduced L and R amygdala volume	—	—

Note.

^1^All studies used healthy adults; — = trait was not studied; also see meta-analysis of negative emotionality (Mincic, 2015).

Additional insights into the amygdala and hippocampus in relation to neuroticism may be gleaned from well-powered meta-analyses of closely associated disorders, major depression and anxiety [7]. Co-morbid major depression and anxiety disorders have been associated with a smaller right amygdala [41], and the most recent meta-analysis of major depressive disorder found it was associated with smaller left hippocampus [40]. Furthermore, a meta-analysis of the morphometry of all common psychiatric disorders found consistent gray matter loss in a number of regions including amygdala and hippocampus [42]. They found this effect was primarily driven by major depressive disorder. Despite these findings, the extent to which decreased hippocampal and amygdalar volume is a cause or consequence of depression or anxiety remains uncertain [40,41,43]. In sum, while not present in every meta-analysis, smaller hippocampus and sometimes amygdala is present in individuals with depression, especially with co-occurring anxiety. This is in peculiar contrast to the finding of a larger left amygdala in relation to negative emotionality, given the established links between neuroticism and internalizing psychopathology [7,35]. Despite this promising work, no large well-powered studies have explored the hippocampus and amygdala in relation to the FFM, with the exception of a single study focusing on the amygdala and neuroticism [36]. As such, lack of statistical power renders most of these findings difficult to interpret, and it is uncertain the extent to which these findings will replicate in adequately powered tests.

There are many possible explanations for the inconsistent findings in the hippocampus and amygdala literature. In particular, relatively small sample sizes of individual studies, namely those of extraversion, may limit the replicability of findings with regard to the amygdala. Additionally, recent advances in software such as FreeSurfer [44] now enable much more reliable whole hippocampal and amygdalar estimation [45,46], and also provide subdivisions of both regions, allowing for a higher resolution. In particular, the dentate gyrus and CA2/3 of the hippocampus have been specifically linked to early life adversity and stress [47–49], two environmental factors that have also been shown to increase neuroticism and decrease conscientiousness and agreeableness over time [50]. However, the research on these subfields in relation to major depressive disorder, a close correlate of neuroticism, has been inconsistent (e.g., [51,52]). Although the amygdalar nuclei are not well-studied in humans, the nuclei, namely the central amygdala, have been well-researched in animal models. This research has found the central amygdala is essential to response to fearful stimuli, stressful stimuli, and drug-related stimuli [53]. Therefore, examining the nuclei of the amygdala in relation to personality may help clarify specific functions in humans.

### Current study

The current investigation leverages recent advances in hippocampal and amygdalar segmentation software [45,46] and the full Human Connectome Project data (N ~ = 1200; https://db.humanconnectome.org/). Specifically, the study assessed the relationship between the whole hippocampus and amygdala and their subdivisions with the FFM using the largest sample to date. Based on prior work, our hypotheses were that reduced gray matter volume (GMV) of the hippocampus would be associated with neuroticism and GMV of the amygdala would be associated with neuroticism, but in uncertain direction given the conflicting findings across negative emotionality and depression/anxiety research [35,36,41,42]. Furthermore, we hypothesized that the CA2/3 and dentate gyrus would account for the relationship of the whole hippocampus with neuroticism. We also hypothesized that reduced GMV of the bilateral amygdala would be associated with extraversion. Finally, we explored all possible associations of the FFM traits with the subdivisions of the hippocampus and amygdala.

## Materials and methods

### Participants

Data were drawn from the publicly available repository of the WU-Minn HCP (http://www.humanconnectome.org/). Structural MRI data were collected from 1,113 participants at Washington University as part of the Human Connectome Project. Informed consent was obtained for all participants (consent procedure and full inclusion/exclusion criteria are detailed in [54,55]). Participants were 22–37 years old and had no significant history of psychiatric disorder, substance abuse, neurological disorder or damage, cardiovascular disease, or Mendelian genetic disease (e.g., cystic fibrosis). The FFM was assessed with the NEO-FFI, a 60-item self-report measure that uses 12 items to assess each FFM domain [14]. 8 participants did not complete the NEO-FFI and thus the final sample comprised 1105 participants (Table 2).

**Table 2:** Demographic characteristics (N = 1105).

	M (SD) or %
Sex	
Male	45.7%
Female	54.3%
Age	28.8 (3.7)
Race	
White or Caucasian	74.8%
Black or African American	15.1%
Asian American, Native Hawaiian, or other Pacific Islander	5.7%
Native American	.2%
More than one race	2.5%
Not sure or unknown	1.7%
Ethnicity	
Hispanic or Latino	8.5%
Not Hispanic or Latino	90.3%
Not sure or unknown	1.2%
Income	
$1,000-$9,999/year	7.1%
$10,000-$19,999/year	7.9%
$20,000-$29,999/year	12.5%
$30,000-$39,999/year	12.0%
$40,000-$49,999/year	10.3%
$50,000-$74,999/year	21.1%
$75,000-$99,999/year	13.5%
$100,000-$149,999/year	15.6%
Years of Education	14.92 (1.80)

### MRI data acquisition and pre-processing

Structural images were collected on a 3T Siemens Skyra scanner (Siemens AG, Erlanger, Germany) with a 32-channel head coil. T1-weighted structural images were acquired with a resolution of 0.7 mm^3^ isotropic (FOV = 224x240, matrix = 320x320, 256 sagittal slices; TR = 2400 ms and TE = 2.14 ms). All structural images were reviewed by a technician immediately following acquisition to ensure scans did not have any significant problems (i.e., artifacts, substantial movement). If problems were found, structural scans were reacquired immediately. Within hours of the initial acquisition, scans were examined by quality control specialists who assessed them for image crispness, blurriness, motion and other artifacts, and accuracy of defacing. Based on these factors, scans were rated on a 1 to 4 scale (poor to excellent). In all cases where structural scans were below 3 (good), new structural scans were reacquired on the participant’s second study day. Through this process, all subjects had high-quality structural imaging data.

Data were reconstructed and preprocessed using Chris Rorden’s DICOM to NIFTI conversion software [56] and a modified version of the FreeSurfer automated “recon-all” pipeline [57–59] in FreeSurfer Image Analysis Suite version 5.3 (http://surfer.nmr.mgh.harvard.edu) [44]. That pre-processing pipeline includes correction of distortions in the raw MR images resulting from several sources, skull-stripping, labeling of tissue types in the brain (i.e., gray matter, white matter, cerebrospinal fluid), identification of sulci and gyri, tessellation of the surface of the brain, and warping of images into a common space [57]. The output of this pipeline is GMV for each region, as well as several summary statistics including total intracranial volume (ICV) which was utilized as a covariate in this study.

### Hippocampus and amygdala segmentation

Hippocampal subfield segmentation was derived using the automated algorithm available in FreeSurfer version 6.0 [45]. This method uses a new atlas of the hippocampus and its nuclei built on a combination of manual annotations of the hippocampal subregions from 15 ultrahigh resolution, ex vivo images and data from an independent set of 39 in vivo, T1-weighted, 1-mm resolution MRI scans. A validation study of this method found the new segmentation procedure to have a high degree of test-retest and transplatform reliability across scanning modalities (1.5-T vs 3-T scanners) [60]. Likewise, amygdala nuclei segmentation was completed utilizing the new automated algorithm available in FreeSurfer version 6.0 [46]. This methodology also utilizes a new atlas of amygdala segments generated from postmortem scans conducted at high resolution and 7T field strength. A recent validation study indicates this new atlas is an improvement over past whole amygdala estimations and effectively discriminates between Alzheimer’s disease and age-matched controls [46]. In the current study, the CA4 and granule cell layer were combined because they are both components of the dentate gyrus and the molecular layer was excluded because it is not easily distinguished in T1-weighted images [45].

The segmentation procedure for the hippocampus and amygdala enables the visualization and quantification of hippocampus amygdala nuclei, which are the subject of the current investigation. These procedures use the newly constructed atlases of the hippocampus and amygdala (described above) to automatically segment the hippocampus and amygdala nuclei from the MRI data of each individual subject. Hippocampus and amygdala segmentation analyses are conducted as an iterative optimization problem in a Bayesian inference framework that attempts to identify the segment to which each voxel is most likely to belong based its recorded intensity and location in the constructed atlases described above [45,46]. The outputs of these analyses are GMV (in mm^3^) for each nuclei of the hippocampus and amygdala (separately for left and right side), as well as for the whole hippocampus and amygdala. Thus, these automated segmentation procedures use Bayesian inference to apply the newly created and validated atlases of the hippocampus and amygdala to the data of novel, *in vivo* subjects in order to determine these individuals’ GMV in the segments described in these atlases.

### Statistical analyses

For preliminary analyses we conducted independent samples t-tests and analyses of covariance (ANCOVAs; controlling for age and ICV) to test for gender differences in FFM traits and amygdala/hippocampal volumes, respectively. To control for type I error rate in primary analyses, we utilized a hierarchical analytical approach based on the level of prior empirical support for the regions with at least one FFM trait. In all cases, we conducted linear regressions with the brain region as the dependent variable and FFM traits as the independent variables. Age, gender, and ICV were also included in all analyses as covariates. At the first stage of FWE correction, we conducted four regressions of the FFM and left and right amygdala and hippocampus. In the second stage, we conducted four regressions of the FFM with the previously supported subdivisions of the hippocampus: the left and right CA2/3 and dentate gyrus (i.e., CA4/GC; CA4 and GC were combined because they are both components of the dentate gyrus and because the ability to distinguish the molecular layer in T1-weighted images is limited^35^). In the third stage, we conducted simultaneous regressions of the FFM and the remaining nuclei and segmentations that have not been explored as thoroughly in humans to date: 1) 18 nuclei of the amygdala (9 on each side); and 2) 14 segmentations of the hippocampus (7 on each side). The false discovery rate (FDR; [61]) was set at *q* < .05, and each level of analysis included the *p*-values from the prior levels (i.e., stage 1 = FDR correction for 4 tests; stage 2 = FDR correction for 8 tests; stage 3 = FDR correction for all 40 tests).

To ensure effects were not missed by eliminating shared variance in regressions including all FFM traits simultaneously (e.g., [62,63]), we conducted separate linear regressions for each of the FFM traits and the left and right amygdala, hippocampus, CA2/3, and dentate gyrus. We also report regressions for the remaining exploratory regions.

To ensure we did not miss gender-specific effects, as has been found in cortical FFM analyses (see [64]), we conducted linear regressions which included covariates, one FFM trait, gender, and a FFM trait by gender interaction term. Finally, we conducted nonlinear regressions with quadratic terms such that each regression included: both linear and quadratic terms for one FFM trait, gender, age, and ICV. These gender interaction and nonlinear regressions were only run for the 4 a priori regions on each side. Both nominal and FDR corrected significance were reported in all analyses.

## Results

### Preliminary analyses

A correlation matrix of the FFM (including internal reliabilities) can be found in Table 3. The FFM were generally correlated as expected [65] and exhibited acceptable to good internal reliability (*α* = .75–84). Generally consistent with prior research of sex differences in personality in the United States [66], women exhibited significantly lower levels of openness; higher levels of agreeableness, conscientiousness, and neuroticism (*p*s < .005); and no differences on extraversion (means and standard deviations in S1 Table). With regard to brain regions, after controlling for age and ICV, women had significantly smaller left and right amygdala and larger left hippocampus (along with several corresponding subfields; S2 Table). Differences in amygdala volume are consistent with the literature [67] whereas differences in hippocampal volume are inconsistent [67,68] and are quite small in this study.

**Table 3:** Means and Pearson correlations among FFM traits.

Variable	M(SD)	1	2	3	4
1. Agreeableness (*α* = .76)	33.5(5.8)	—-	—-	—-	—-
2. Openness (*α* = .75)	28.3(6.2)	.09*	—-	—-	—-
3. Conscientiousness (*α* = .82)	34.5(5.9)	.23*	-.13*	—-	—-
4. Neuroticism (*α* = .84)	16.6(7.4)	-.29*	.01	-.40*	—-
5. Extraversion (*α* = .77)	30.7(6.0)	.28*	.10*	.26*	-.35*

Note.

* = *p* < .005

### Whole amygdala and hippocampus

In the linear regressions containing covariates (i.e., age, gender, and ICV) and the FFM, no nominally significant relationships were found with left or right whole amygdala and hippocampus (Table 4). The strongest but non-significant relationship was found between the right amygdala and extraversion (*β* = .04, *p* = .08). However, even when allowing for shared variance among the traits by conducting linear regressions separately for each FFM trait, there were no significant associations between the FFM and these 4 regions (*p*s > .05; S3 Table). Additionally, there were no significant gender by FFM interactions.

**Table 4:** Univariate linear regressions of the whole amygdala, whole hippocampus, CA2/3, and dentate gyrus with the FFM and age, gender, and ICV included as covariates. For each variable β(p).

	Agreeableness	Openness	Conscientiousness	Neuroticism	Extraversion
L amygdala	.01(.68)	.00(.85)	-.02(.34)	.02(.57)	.03(.25)
R amygdala	.04(.13)	.00(.88)	-.02(.38)	.03(.22)	.04(.08)
L hippocampus	-.02(.62)	.01(.69)	.00(.96)	-.01(.88)	.04(.22)
R hippocampus	-.02(.65)	.01(.78)	.01(.84)	-.02(.53)	.04(.21)
L CA2/3	-.01(.76)	.03(.38)	-.01(.72)	.00(.90)	.02(.49)
R CA2/3	-.01(.73)	.00(.89)	-.03(.36)	-.04(.26)	.04(.24)
L dentate gyrus	-.02(50)	.01(.79)	.00(.94)	.02(.60)	.03(.43)
R dentate gyrus	.00(.95)	.00(.96)	-.01(.69)	-.02(.50)	.03(.31)

Note. ICV = intracranial volume.

In the quadratic regressions, the squared agreeableness term was associated with left and right whole hippocampus (*β* = .50, *p* = .03; *β* = .65, *p* = .01), indicating that a convex relationship exists, such that high and low values of agreeableness are associated with a larger hippocampus. These results were not FDR significant (accounting for 40 tests [8 regions and 5 traits]).

### CA2/3 and dentate gyrus

In the 4 linear regressions of FFM predicting the left and right CA2/3 and dentate gyrus, no nominally significant relationships were found (*p*s > .05; Table 4). Similarly, no additional associations were identified in the linear regressions conducted separately for each FFM trait (*p*s > .05; S2 Table). There was one nominally significant interaction of gender and agreeableness predicting left CA2/3 (*β* = .20, *p* = .048), but this was not FDR significant. Probing the interaction revealed opposite direction of effects by gender, neither of which was nominally significant.

In the quadratic regressions, the squared agreeableness term was associated with left and right dentate gyrus (*β* = .48, *p* = .04, *β* = .67, *p* = .004), indicating that a convex relationship exists, such that high and low values of agreeableness are associated with a larger dentate gyrus. These results were not FDR significant (accounting for 40 tests [8 regions and 5 traits]).

### Exploratory analysis of segmentations of amygdala and hippocampus

With regard to the amygdala, there were several nominal associations with FFM traits (*p* < .05), however, none of these survive FDR correction (see Table 5). Of note, the trend level positive association between right whole amygdala and extraversion noted above appears to be attributable to associations with right accessory basal nucleus (*β* = .05; *p* = .04), anterior amygdaloid area (*β =* .06; *p* = .04), and central nucleus (*β* = .07; *p* = .02). None of the 7 remaining segmentations on the left or right side of the hippocampus were nominally significantly associated with the FFM. There were no additional associations identified in the linear regressions conducted separately for each FFM trait (S3 Table).

**Table 5:** Univariate linear regressions of the remaining nuclei of the amygdala and segmentations of the hippocampus with the FFM and age, gender, and ICV included as covariates. For each variable β(p).

	Agreeableness	Openness	Conscientiousness	Neuroticism	Extraversion
**Amygdala**					
L lateral nucleus	.02(.43)	-.01(.60)	-.02(.37)	.03(.27)	.03(.20)
R lateral nucleus	.03(.33)	-.01(.60)	-.03(.23)	.02(.57)	.04(.16)
L basal nucleus	.03(.29)	.00(.93)	-.03(.18)	.01(.65)	.02(.45)
R basal nucleus	**.06(.02)**	.01(.62)	-.03(.23)	.04(.13)	.03(.22)
L Ac basal nucleus	-.01(.60)	.01(.79)	-.02(.47)	-.01(.84)	.02(.37)
R Ac basal nucleus	.03(.30)	.00(.96)	-.01(.83)	.03(.32)	**.05(.04)**
L An amygdaloid area	.00(.92)	-.01(.80)	-.02(.50)	.02(.58)	.03(.24)
R An amygdaloid area	.04(.15)	-.01(.58)	-.01(.73)	.04(.20)	**.06(.04)**
L central nucleus	-.01(.78)	-.01(.59)	.02(.60)	.05(.14)	.05(.11)
R central nucleus	.01(.83)	-.02(.40)	.02(.61)	.05(.13)	**.07(.02)**
L medial nucleus	-.04(.27)	.01(.76)	-.00(.89)	-.04(.24)	.02(.53)
R medial nucleus	-.05(.09)	.01(.75)	.04(.18)	-.02(.47)	.04(.26)
L cortical nucleus	-.04(.17)	.03(.28)	.01(.66)	-.02(.52)	.01(.68)
R cortical nucleus	-.02(.46)	.01(.80)	.03(.33)	.00(.98)	.04(.24)
L corticoamygdaloid T	-.01(.63)	-.01(.64)	-.01(.68)	.00(.91)	.02(.42)
R corticoamygdaloid T	.05(.10)	-.01(.75)	-.01(.77)	**.06(.04)**	.04(.17)
L paralaminar nucleus	**.06(.03)**	.01(.82)	-.03(.21)	.00(.95)	-.01(.76)
R paralaminar nucleus	**.06(.02)**	.02(.45)	-.05(.07)	.02(.55)	.00(.99)
**Hippocampus**					
L subiculum	.00(.93)	.02(.61)	.02(.61)	-.02(.54)	.05(.17)
R subiculum	-.03(.44)	.02(.64)	.02(.67)	-.01(.69)	.05(.18)
L presubiculum	.00(.99)	.01(.64)	-.02(.65)	.01(.83)	.04(.20)
R presubiculum	-.02(.52)	.01(.83)	.03(.41)	.02(.67)	.02(.53)
L parasubiculum	.02(.52)	.01(.69)	-.01(.84)	.04(.24)	.05(.11)
R parasubiculum	.00(.96)	.00(.93)	.04(.29)	.02(.53)	-.01(.87)
L CA1	.00(.97)	.01(.66)	.01(.83)	.03(.46)	.03(.31)
R CA1	-.01(.72)	.02(.48)	.01(.72)	-.01(.84)	.04(.29)
L fimbria	.02(.57)	-.02(.62)	.01(.77)	-.05(.15)	-.04(.24)
R fimbria	-.01(.88)	.00(.99)	.04(.29)	-.02(.62)	-.06(.08)
L HATA	-.03(.44)	.02(.56)	.03(.43)	-.02(.65)	.02(.47)
R HATA	-.02(.51)	-.01(.76)	.03(.40)	.00(.97)	.04(.25)
L hippocampal fissure	.07(.05)	.03(.39)	-.02(.48)	.02(.66)	-.01(.71)
R hippocampal fissure	.00(.98)	.05(.15)	.04(.24)	.02(.62)	.03(.42)

Note. ICV = intracranial volume, Ac = accessory, An = anterior, T = transition, HATA = hippocampal-amygdaloid transition area. Bolding indicates nominal significance (*p* < .05).

## Discussion

This study explored the amygdalar and hippocampal correlates of the FFM. Based on previous research, we hypothesized: 1) reduced GMV of the hippocampus would be associated with neuroticism and GMV of the amygdala would be associated with neuroticism, but with no hypothesized direction; 2) CA2/3 and dentate gyrus would account for the relationship of the hippocampus with neuroticism, and 3) reduced GMV of the amygdala would be associated with extraversion. In contrast to prior findings, we did not find any significant relationships among these regions, and this was consistent between univariate and multivariate models of personality predicting amygdalar or hippocampal GMV. Our exploratory analyses of the FFM traits and the subdivisions of the hippocampus and amygdala also suggested no significant relationships.

These three sets of hypotheses were grounded in prior work on the FFM traits and psychopathology closely associated with neuroticism (i.e., major depressive disorder and generalized anxiety disorder). Specifically, well-powered work on depression and anxiety generally found smaller hippocampus and sometimes amygdala [40–42], whereas a previous activation likelihood estimation (ALE) meta-analysis of negative emotionality found an association with larger amygdala [35]. With regard to the specific studies on the NEO-FFI measured neuroticism, one identified larger bilateral amygdala [36], one identified a larger volume in the left amygdala [31], but five found no association [28,29,32–34] (Table 1). The largest single study (N = 1050) used similar covariates (age, sex, ICV; they also included IQ) and population (i.e., healthy young adults) to the present investigation, but did find a positive association between bilateral amygdala GMV and neuroticism. It may be that the failure to replicate is attributable to improvements made to amygdala segmentation since their investigation [46]. Additionally, if a true relationship existed between neuroticism and amygdalar GMV, it would be expected that a closer look at subnuclei of the amygdala would yield a relationship, but none was found here. With regard to the hippocampus and neuroticism, only one study was completed to date and it found no association [34]. Research was similarly inconsistent with regard to extraversion and the amygdala, with two studies finding associations in opposite directions [29,30], and five not finding any [28,31–34]. The current investigation did not replicate any positive findings despite using the largest sample to date, the most accurate hippocampal and amygdalar segmentation methods to date [45,46], and assessing subregions,gender interactive effects, and nonlinear effects.

With regard to the exploratory analyses, given the role of both the traits and the hippocampus and amygdala in socioemotional functioning [22,23,37], we anticipated some significant associations among the whole structures or their substructures. While we did find nominally significant associations among some select traits and amygdalar subnuclei, none of these survived false discovery rate correction. Given sex differences in the FFM and in the biology of depression [69,70], we explored gender-specific findings, but identified no differences in effects sizes across any of the traits and a priori regions. There were some nominal associations of nonlinear relationships between agreeableness and hippocampus and dentate gyrus that will need to be followed up on in future research. Given the null findings of the whole hippocampus and amygdala and their subregions with FFM traits, there is little evidence of an association in a relatively healthy young sample of the largest size to date.

This study was not without its limitations. The study was comprised of a relatively healthy population [55], broadly defined, and therefore it is limited in its ability to generalize to more severe clinical samples. However, the sample includes notable levels of anxiety and depressive symptoms [71], and Riccelli et al. (2017) identified numerous cortical associations in the same sample, but half the size. Furthermore, healthy samples were used in the prior studies of neuroticism, extraversion, and conscientiousness with the amygdala and hippocampus. Nonetheless, it will be important for future investigations to include more severe clinical samples to assess for consistency of findings. A second limitation to generalizability is this sample was a relatively tight age range (years 22–37) and therefore generalization to youth and older adults is uncertain, particularly given the relationship of aging with hippocampal size [72].

In the largest study of the FFM and structural morphometry to date, using the highest resolution segmentation methodology available, no associations between FFM traits and amygdalar and hippocampal volumes were found. These null findings are consistent with a recent methodological paper that found small studies are at a high risk for overestimating the effect sizes of brain-behavior correlations [73,74]. Nonetheless, these regions appear to be functionally implicated in personality and future investigations should continue to parse these relationships in unique samples and via diffusion tensor imaging and relevant fMRI tasks (e.g., [75–77]).

## Supporting information

S1 Table Independent samples t-tests of FFM separated by gender. (DOCX)

S2 Table Mean (SD) gray matter volume of amygdala and hippocampus subregions in mm^3^ and ANCOVAs of differences between males and females controlling for age and ICV (means reported in the table are unadjusted). (DOCX)

S3 Table Univariate linear regressions of the amygdala, hippocampus, CA2/3, and dentate gyrus with one trait from the FFM and age, gender, and ICV included as covariates. For each variable β(p).
(DOCX)

S4 Table Univariate linear regressions of the remaining nuclei of the amygdala and segmentations of the hippocampus with one trait from the FFM and age, gender, and ICV included as covariates. For each variable β(p).
(DOCX)

S5 Table Quadratic regressions of the whole amygdala, whole hippocampus, CA2/3, and dentate gyrus with one trait from the FFM and age, gender, and ICV included as covariates. For each variable β(p) of the quadratic term (X^2^) is reported.
(DOCX)
