# Supplementary material for: No evidence for morphometric associations of the amygdala and hippocampus with the five-factor model personality traits in relatively healthy young adults
Source: PLoS One. 2018 Sep 20;13(9):e0204011. doi: 10.1371/journal.pone.0204011 (PMC6147458; doi:10.1371/journal.pone.0204011)
Supplement: S1 Table — (DOCX) [file pone.0204011.s001.docx]

S1 Table

Independent samples t-tests of FFM separated by gender.

|  | Males | Females |  |
| --- | --- | --- | --- |
|  | M(SD) | M(SD) | *p* |
| **Agreeableness** | **32.0(5.8)** | **34.8(5.5)** | **<.001** |
| **Openness** | **28.9(6.3)** | **27.8(6.1)** | **.003** |
| **Conscientiousness** | **33.8(5.9)** | **35.1(5.8)** | **<.001** |
| **Neuroticism** | **15.7(7.7)** | **17.4(7.0)** | **<.001** |
| Extraversion | 30.8(6.0) | 30.7(5.9) | .833 |

Note. Bolding indicates significance (*p* < .05).
