# Supplementary material for: No evidence for morphometric associations of the amygdala and hippocampus with the five-factor model personality traits in relatively healthy young adults
Source: PLoS One. 2018 Sep 20;13(9):e0204011. doi: 10.1371/journal.pone.0204011 (PMC6147458; doi:10.1371/journal.pone.0204011)
Supplement: S2 Table — (DOCX) [file pone.0204011.s002.docx]

S2 Table

Mean (SD) gray matter volume of amygdala and hippocampus subregions in mm^3^ and ANCOVAs of differences between males and females controlling for age and ICV (means reported in the table are unadjusted).

| Region | Total | Male  (n = 505) | Female  (n=600) | ANCOVA  *p*-value |
| --- | --- | --- | --- | --- |
| **Amygdala** |  |  |  |  |
| L whole amygdala* | 1882.0(221.1) | 2016.1(204.4) | 1769.2(164.4) | **<.001** |
| R whole amygdala* | 1940.1(218.6) | 2070.1(195.6) | 1830.6(172.2) | **<.001** |
| L lateral nucleus* | 685.6(83.9) | 735.7(77.1) | 643.3(63.9) | **<.001** |
| R lateral nucleus* | 728.9(86.5) | 777.8(79.0) | 687.6(69.3) | **<.001** |
| L basal nucleus* | 485.6(60.0) | 520.9(56.5) | 455.9(44.9) | **<.001** |
| R basal nucleus* | 499.4(59.5) | 533.6(54.1) | 470.7(47.5) | **<.001** |
| L Ac basal nucleus* | 288.7(37.1) | 309.3(36.1) | 271.3(27.8) | **<.001** |
| R Ac basal nucleus* | 287.3(35.5) | 306.8(33.1) | 271.0(28.6) | **<.001** |
| L An amygdaloid area* | 65.0(09.2) | 69.1(9.1) | 61.6(7.7) | **<.001** |
| R An amygdaloid area* | 70.4(09.7) | 75.3(9.3) | 66.2(8.0) | **<.001** |
| L central nucleus* | 48.4(09.4) | 52.6(9.4) | 44.9(7.8) | **<.001** |
| R central nucleus* | 57.2(10.4) | 61.4(10.2) | 53.6(9.1) | **<.001** |
| L medial nucleus* | 23.5(06.0) | 25.5(6.4) | 21.8(5.1) | **<.001** |
| R medial nucleus* | 24.5(06.3) | 26.4(6.6) | 22.9(5.6) | **<.001** |
| L cortical nucleus* | 27.4(04.9) | 29.4(5.2) | 25.7(3.9) | **<.001** |
| R cortical nucleus* | 25.9(04.8) | 27.8(5.0) | 24.3(4.0) | **<.001** |
| L corticoamygdaloid T* | 204.0(26.0) | 216.1(26.3) | 193.9(20.9) | **<.001** |
| R corticoamygdaloid T* | 193.4(23.5) | 204.5(22.5) | 184.0(19.9) | **<.001** |
| L paralaminar nucleus* | 53.8(06.7) | 57.5(6.4) | 50.7(5.2) | **<.001** |
| R paralaminar nucleus* | 53.1(06.3) | 56.5(5.9) | 50.2(5.3) | **<.001** |
| **Hippocampus** |  |  |  |  |
| L whole hippocampus* | 3692.7(389.3) | 3670.1(393.6) | 3711.8(385.0) | .012 |
| R whole hippocampus | 3707.7(370.4) | 3695.0(376.7) | 3718.4(364.9) | .131 |
| L CA2/3 | 222.4(29.3) | 221.5(29.5) | 223.3(29.2) | .087 |
| R CA2/3 | 235.1(30.3) | 235.0(30.6) | 235.2(30.2) | .910 |
| L dentate gyrus* | 310.9(35.4) | 308.6(35.9) | 312.9(34.9) | .005 |
| R dentate gyrus | 315.6(33.9) | 314.6(34.7) | 316.4(33.2) | .308 |
| L subiculum | 458.2(53.6) | 456.1(55.2) | 459.9(52.2) | .089 |
| R subiculum | 451.5(50.0) | 450.5(51.1) | 452.3(49.1) | .246 |
| L presubiculum | 458.2(53.6) | 337.3(42.7) | 340.9(42.5) | .040 |
| R presubiculum* | 451.5(50.0) | 320.1(39.8) | 324.1(39.7) | .019 |
| L parasubiculum | 78.5(13.0) | 78.6(13.2) | 78.5(12.9) | .308 |
| R parasubiculum | 77.8(13.2) | 77.3(13.3) | 78.2(13.1) | .094 |
| L CA1* | 692.8(84.4) | 688.4(85.2) | 696.5(83.7) | .027 |
| R CA1 | 716.0(82.3) | 714.6(83.3) | 717.2(81.5) | .261 |
| L fimbria* | 99.1(19.9) | 98.0(19.8) | 100.1(19.9) | .009 |
| R fimbria* | 96.1(19.0) | 95.3(19.4) | 96.7(18.7) | .031 |
| L HATA | 71.4(10.7) | 70.8(10.6) | 71.9(10.7) | .036 |
| R HATA | 68.1(09.9) | 67.7(9.9) | 68.4(9.9) | .100 |
| L hippocampal fissure | 139.7(22.2) | 139.8(23.0) | 139.6(21.6) | .985 |
| R hippocampal fissure | 146.7(22.2) | 146.2(22.6) | 147.1(21.9) | .505 |

Note. Bolding indicates nominally significant (*p* < .05) and * indicates false discovery rate correction significant (q < .05; adjusting for 40 tests).
