# Supplementary material for: No evidence for morphometric associations of the amygdala and hippocampus with the five-factor model personality traits in relatively healthy young adults
Source: PLoS One. 2018 Sep 20;13(9):e0204011. doi: 10.1371/journal.pone.0204011 (PMC6147458; doi:10.1371/journal.pone.0204011)
Supplement: S3 Table — For each variable β(p). (DOCX) [file pone.0204011.s003.docx]

S3 Table

Univariate linear regressions of the amygdala, hippocampus, CA2/3, and dentate gyrus with one trait from the FFM and age, gender, and ICV included as covariates. For each variable β(p).

|  | Agreeableness | Openness | Conscientiousness | Neuroticism | Extraversion |
| --- | --- | --- | --- | --- | --- |
| L amygdala | .01(.71) | .00(.90) | -.02(.37) | .01(.60) | .02(.39) |
| R amygdala | .03(.13) | .01(.72) | -.02(.48) | .01(.52) | .04(.10) |
| L hippocampus | .00(.97) | .02(.62) | .01(.83) | -.01(.65) | .04(.20) |
| R hippocampus | .01(.82) | .01(.75) | .02(.44) | -.04(.25) | .05(.11) |
| L CA2/3 | .00(.96) | .03(.33) | -.01(.75) | .00(.91) | .02(.49) |
| R CA2/3 | .01(.85) | .00(.97) | -.01(.84) | -.04(.22) | .04(.18) |
| L dentate gyrus | -.02(.53) | .01(.78) | .00(.89) | .02(.60) | .02(.63) |
| R dentate gyrus | .01(.67) | .00(.92) | .01(.86) | -.03(.32) | .04(.20) |

Note. ICV = intracranial volume. Separate linear regressions were conducted for each of the FFM traits. Bolding indicates nominal significance (*p* < .05).
