# Supplementary material for: No evidence for morphometric associations of the amygdala and hippocampus with the five-factor model personality traits in relatively healthy young adults
Source: PLoS One. 2018 Sep 20;13(9):e0204011. doi: 10.1371/journal.pone.0204011 (PMC6147458; doi:10.1371/journal.pone.0204011)
Supplement: S4 Table — For each variable β(p). (DOCX) [file pone.0204011.s004.docx]

S4 Table

Univariate linear regressions of the remaining nuclei of the amygdala and segmentations of the hippocampus with one trait from the FFM and age, gender, and ICV included as covariates. For each variable β(p).

|  | Agreeableness | Openness | Conscientiousness | Neuroticism | Extraversion |
| --- | --- | --- | --- | --- | --- |
| **Amygdala** |  |  |  |  |  |
| L lateral nucleus | .01(.56) | .00(.88) | -.02(.35) | .02(.35) | .02(.37) |
| R lateral nucleus | .02(.34) | .00(.93) | -.02(.35) | .01(.73) | .03(.23) |
| L basal nucleus | .02(.35) | .01(.62) | -.03(.20) | .01(.62) | .01(.55) |
| R basal nucleus | **.05(.04)** | .03(.27) | -.03(.20) | .02(.28) | .03(.25) |
| L Ac basal nucleus | -.01(.74) | .01(.69) | -.01(.55) | .00(.95) | .02(.48) |
| R Ac basal nucleus | .03(.17) | .01(.72) | .00(.92) | .00(.91) | **.05(.03)** |
| L An amygdaloid area | .00(.93) | .00(.99) | -.02(.52) | .01(.63) | .02(.38) |
| R An amygdaloid area | .04(.10) | .00(.94) | .00(1.00) | .01(.74) | **.05(.04)** |
| L central nucleus | -.01(.77) | -.01(.65) | .01(.74) | .03(.34) | .03(.25) |
| R central nucleus | .01(.64) | -.02(.54) | .02(.49) | .01(.62) | **.06(.03)** |
| L medial nucleus | -.02(.59) | .01(.82) | .01(.74) | -.03(.24) | .02(.41) |
| R medial nucleus | -.03(.40) | .00(.97) | .05(.07) | -.04(.18) | .04(.15) |
| L cortical nucleus | -.03(.38) | .03(.37) | .01(.61) | -.02(.53) | .01(.60) |
| R cortical nucleus | .00(.88) | .01(.87) | .04(.21) | -.02(.50) | .04(.17) |
| L corticoamygdaloid T | -.01(.64) | -.01(.69) | -.01(.73) | .01(.86) | .01(.61) |
| R corticoamygdaloid T | .04(.17) | .00(.91) | -.01(.61) | .04(.16) | .03(.28) |
| L paralaminar nucleus | **.05(.045)** | .02(.52) | -.02(.28) | .00(.98) | .00(.99) |
| R paralaminar nucleus | .05(.06) | .03(.19) | -.05(.06) | .02(.44) | .00(.99) |
| **Hippocampus** |  |  |  |  |  |
| L subiculum | .02(.59) | .02(.47) | .00(.95) | -.03(.33) | .05(.10) |
| R subiculum | .00(.93) | .01(.64) | .03(.40) | -.03(.36) | .05(.11) |
| L presubiculum | .01(.77) | .02(.49) | -.01(.77) | .00(.98) | .04(.22) |
| R presubiculum | -.01(.66) | .00(.92) | .02(.46) | .00(.92) | .02(56) |
| L parasubiculum | .02(.43) | .02(.48) | -.01(.82) | .02(.53) | .04(.14) |
| R parasubiculum | .00(.92) | -.01(.81) | .03(.40) | .01(.75) | .00(.89) |
| L CA1 | .01(.84) | .02(.58) | .00(.90) | .01(.71) | .03(.35) |
| R CA1 | .01(.84) | .02(.46) | .02(.52) | -.02(.49) | .04(.19) |
| L fimbria | .03(.42) | -.02(.53) | .03(.38) | -.05(.11) | -.01(.63) |
| R fimbria | -.01(.77) | -.01(.70) | .03(.37) | -.01(.72) | -.04(.15) |
| L HATA | -.01(.87) | .01(.64) | .03(.28) | -.03(.36) | .03(.29) |
| R HATA | -.01(.84) | -.01(.70) | .04(.25) | -.02(.56) | .04(.21) |
| L hippocampal fissure | .06(.08) | .04(.24) | -.02(.43) | .01(.74) | .00(.92) |
| R hippocampal fissure | .02(.63) | .04(.15) | .03(.26) | -.01(.81) | .04(.24) |

Note. ICV = intracranial volume, Ac = accessory, An = anterior, T = transition, HATA = hippocampal-amygdaloid transition area. Separate linear regressions were conducted for each of the FFM traits. Bolding indicates nominal significance (*p* < .05).
