# Supplementary material for: No evidence for morphometric associations of the amygdala and hippocampus with the five-factor model personality traits in relatively healthy young adults
Source: PLoS One. 2018 Sep 20;13(9):e0204011. doi: 10.1371/journal.pone.0204011 (PMC6147458; doi:10.1371/journal.pone.0204011)
Supplement: S5 Table — For each variable β(p) of the quadratic term (X2) is reported. (DOCX) [file pone.0204011.s005.docx]

S5 Table.

Quadratic regressions of the whole amygdala, whole hippocampus, CA2/3, and dentate gyrus with one trait from the FFM and age, gender, and ICV included as covariates. For each variable β(p) of the quadratic term (X^2^) is reported.

|  | Agreeableness | Openness | Conscientiousness | Neuroticism | Extraversion |
| --- | --- | --- | --- | --- | --- |
|  | X^2^ | X^2^ | X^2^ | X^2^ | X^2^ |
| L amygdala | -.29(.09) | -.16(.29) | -.13(.46) | .00(.96) | -.08(.61) |
| R amygdala | -.06(.71) | .08(.61) | -.21(.24) | .03(.68) | -.26(.09) |
| L hippocampus | **.50(.03)** | .30(.16) | -.04(.88) | .02(.87) | -.19(.38) |
| R hippocampus | **.65(.01)** | .20(.35) | -.06(.80) | .03(.76) | -.08(.71) |
| L CA2/3 | .16(.51) | .20(.34) | .24(.32) | -.11(.31) | -.28(.19) |
| R CA2/3 | .22(.36) | .12(.56) | .21(.39) | -.08(.48) | -.19(.38) |
| L dentate gyrus | **.48(.04)** | .24(.26) | -.02(.95) | .01(.94) | -.32(.13) |
| R dentate gyrus | **.67(.004)** | .13(.54) | -.04(.88) | .02(.85) | -.13(.54) |

Note. ICV = intracranial volume. Bolding indicates nominal significance (*p* < .05).
